# Geometric Modeling of Cellular Materials for Additive Manufacturing in Biomedical Field: A Review

**DOI:** 10.1155/2018/1654782

**Published:** 2018-02-01

**Authors:** Gianpaolo Savio, Stefano Rosso, Roberto Meneghello, Gianmaria Concheri

**Affiliations:** ^1^Department of Civil, Environmental and Architectural Engineering, Laboratory of Design Tools and Methods in Industrial Engineering, University of Padova, Via Venezia 1, 35131 Padova, Italy; ^2^Department of Management and Engineering, Laboratory of Design Tools and Methods in Industrial Engineering, University of Padova, Stradella S. Nicola 3, 36100 Vicenza, Italy

## Abstract

Advances in additive manufacturing technologies facilitate the fabrication of cellular materials that have tailored functional characteristics. The application of solid freeform fabrication techniques is especially exploited in designing scaffolds for tissue engineering. In this review, firstly, a classification of cellular materials from a geometric point of view is proposed; then, the main approaches on geometric modeling of cellular materials are discussed. Finally, an investigation on porous scaffolds fabricated by additive manufacturing technologies is pointed out. Perspectives in geometric modeling of scaffolds for tissue engineering are also proposed.

## 1. Introduction

Since the introduction of rapid prototyping (RP) in the late 1980s [1], additive manufacturing (AM) received an increasing interest from the scientific research community. According to ISO/ASTM standards [2], AM is defined as “a process of joining materials to make objects from 3D model data, usually layer upon layer, as opposed to subtractive manufacturing methodologies.” AM processes are categorized into seven groups: binder jetting, directed energy deposition, material extrusion, material jetting, powder bed fusion, sheet lamination, and vat photopolymerization. The fundamentals of these processes can be found in the literature [3]. Thanks to this layer manufacturing approach, it is possible to fabricate geometrically complex parts which could not be realized at all with a conventional manufacturing process. Moreover, Holmström et al. [4] highlight other benefits of AM if compared to conventional ones:

- No tooling needed, reducing production ramp-up time and cost
- Small production batches which are feasible and economical
- Possibility to quickly change design
- Possibility to optimize products for function
- Possibility to reduce waste
- Potential for simpler supply chains, shorter lead times, and lower inventories
- Design customization

In this context, Rosen [5] drew attention to the need of introducing the new concept of design for additive manufacturing (DFAM) defined as the “synthesis of shapes, sizes, geometric mesostructures, and material compositions and microstructures to best utilize manufacturing process capabilities to achieve desired performance and other life-cycle objectives”. In this framework, the Process-Structure–Property-Behavior model was adopted where each item represents an object data field with its own properties and features; the traversal from behavior to process can be called design, while the reverse direction can be called analysis. Another DFAM methodology [6] is organized into three main steps; the first step consists of determining the functional surface position of the studied design problem into the manufacturing machine to determine the design area and it is essential in order to have a trade-off between quality and cost. The second step deals with functional optimization, and a numerical optimization approach is used, namely, topological optimization. The third step is needed to determine the optimized manufacturing paths as a function of the manufacturing process characteristics. Moreover, Boyard et al. [7] proposed a DFAM at a product level, taking into consideration the assembly relationship between parts in a complex product with consideration of both functionality and manufacturing, instead of limiting the design at a single part level. This is done by generating a graph of functions coming from functional specifications, where every product part is represented by a set; the graph organization allows to perform the architectural design phase (DFA) simultaneously with the detailed design phase (DFM). An extensive review on DFAM proposes an overview of classical design for manufacturing and assembly (DfMA) and examines the suitability of that definition and framework for AM applications [8].

In this scenario, AM allows to fully exploit cellular solids. These materials found several applications in biomedical fields, especially in the scaffold design for tissue engineering based on additive manufacturing technologies. However, literature highlights limits and constraints associated with CAD tools, discretization, directionality, appropriate building orientation, need for support, material properties, process characteristics, metrology, quality control, maintenance, and regulatory [8].

In this work, a novel cellular material classification is proposed and then a review on geometric modeling techniques is presented. Finally, applications in a scaffold design for tissue engineering in the biomedical field are stated highlighting specific geometric modeling methods proposed in the literature.

## 2. Cellular Materials

According to the definition proposed by Gibson and Ashby, a cellular solid is a material “made up of an interconnected network of solid struts or plates which form the edges and faces of cells”; random structures such as sponges and cork are included too [9]. Due to the appearance of the obtained material, cellular solids are usually referred to as lattice structures. The typical dimension of a unit cell is between 0.1 and 10 mm, that is, at a mesoscale level; larger features than mesoscale are counted as macroscale, while smaller ones are counted as microscale [10].

Adopting additive manufacturing technologies, the single cell can be designed at will, so the material is placed only where it is needed for a specific application. Consequently, a lattice structure has many superior properties: it is lightweight in relation to its high specific stiffness and strength; it is a good heat exchanger due to its large surface area and a good energy absorber due to its ability to undergo large deformation at a relatively low stress level and ensure acoustic insulation due to its large number of internal pores.

Recently, Amin Yavari et al. [11] proposed to use the term “metamaterials” when dealing with microscale unit cells; according to them, a metamaterial can be considered a structure as far as its small-scale features and properties are concerned, and at the same time, it behaves like a homogeneous material when macroscopic properties are analyzed. This definition slightly differs compared to the one given by Kshetrimayum, where metamaterials are artificial materials with unusual electromagnetic properties that are not found in naturally occurring materials [12].

### 2.1. Cellular Material Taxonomy

In literature, several classifications can be found. Tang et al.'s subdivision [13] is based on unit cell organization and geometry. Disordered cellular structures, where lattice units are of different sizes and shapes and are randomly distributed, are distinguished from periodic and pseudoperiodic ones; the former are a simple repetition in space of a single object (the unit cell), and the latter can have the shape changed in space according to specific design purposes. In a successive work [10], they present three different classification methods. The first one is the same as the previous work. The second deals with geometric configuration of each cellular unit and considers foam structures, 2D lattice structures (honeycomb), and 3D lattice structures. The third deals with the deformation criteria and distinguishes between bending-dominated and stretching-dominated cellular structures. A stretch-dominated architecture has a higher modulus and yield strength compared to a bending-dominated architecture with the same relative density; for these reasons, the former is suitable for a lightweight structure design, while the latter is suitable for energy absorption applications [14].

**Figure 1:**
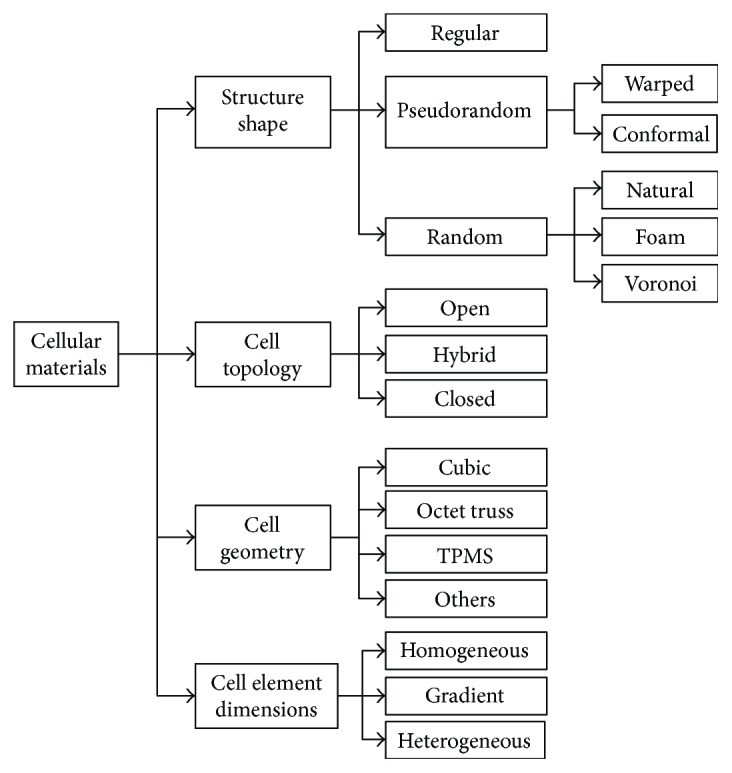
Cellular material classification.

The remaining part of this section will introduce and exhaustively describe an original classification scheme based on the geometry of cellular materials with regard to the distribution of the cells in the whole structure, the cell topology and geometry, and the cell element dimensions (Figure 1).

**Figure 2:**
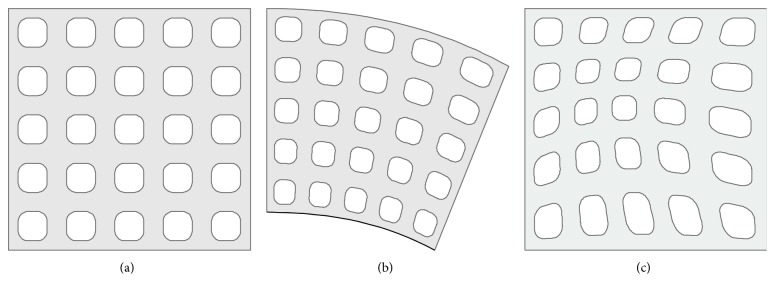
Exemplification of a (a) regular, (b) conformal (the structure is bended on the thick curve), and (c) warped cellular materials.

**Figure 3:**
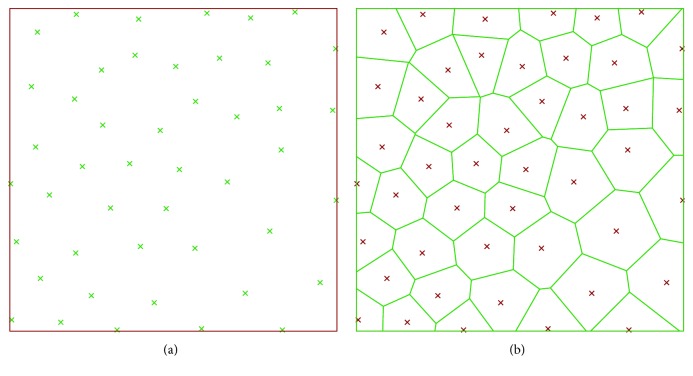
Random cellular material in 2D: (a) seed and (b) region partitioning.

At a structure shape level, cellular materials can be divided into regular, pseudorandom, and random structures (Figures 2 and 3). *Regular* cellular materials consist in a simple repetition of the unit cell in the entire design volume. *Pseudorandom* structures are obtained maintaining the topology and varying both size and geometry. These cellular materials can be further divided into warped and conformal structures. *Warped* structures are realized deforming the unit cell, keeping the original topology; there are several deformation pattern possibilities, for example, according to FEA (e.g., structural, fluid dynamics, or thermal), position with respect to a reference frame, and other functional requirements. In c*onformal* structures, the geometry and size of each cell are different in order to adapt (i.e., conform) to the external shape of the model. For instance, in Figure 2(b), the regular lattice is fitted to the thick line. Compared to regular cellular materials, conformal ones never present interrupted or incomplete cells; this feature eliminates weakness at boundaries and provides stiffness and resistance to the entire model [15].

*Random* cellular materials concern another branch of the shape of the cellular materials; these structures present a random organization of cells, and the geometry and dimension of cells vary randomly too. In nature, several types of random structures can be found, for example, cork (material), sponge (aquatic animal), and trabecular or cancellous bones [16]. *Foam* structures are obtained using traditional fabrication methods such as gas injection into a metal melt, vapor deposition, or spray foaming [17]; the shape and size of the pores can be partially controlled by changing the parameters of these processes. Foam structures can also be obtained by additive manufacturing technologies using mathematical functions [18] or Boolean operations [19] that allow a full control of the pores. Furthermore, random cellular materials can be modeled adopting a Voronoi diagram, randomly positioning a set of points (seeds) inside the design volume, partitioning the space in regions based on the distance among points and then assigning a thickness to the edges of the regions [20, 21]. An example of a 2D Voronoi diagram is shown in Figure 3, where every cell is the subset in the plane containing the points that are closer to a specific seed than to any other.

Regardless of the structure shape, cellular materials can be classified according to the cell topology. An *open* cellular material presents only cells having an open porous structure which means that the pores are accessible by a fluid. Conversely, if the pores are inaccessible, the element is called *closed* cellular material; when compared to an open one, this structure offers more stiffness but, at the same time, hinders fluid exchange and prevents the emptying of the material in the additive manufacturing process. If a model presents parts with open cells and parts with closed cells, it can be referred as *hybrid* cell topology cellular material. Open cellular materials are preferred in biomedical applications, such as tissue engineering, where the connection between cells are needed to allow fluid exchange and tissue regrowth, while closed cellular materials are used for structural purposes.

**Figure 4:**
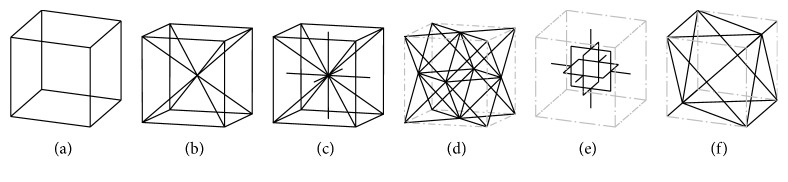
Cell types: (a) SC [22], (b) BCC [22], (c) RBCC [22], (d) OT [23], (e) GA [25], and (f) WG [26].

**Figure 5:**
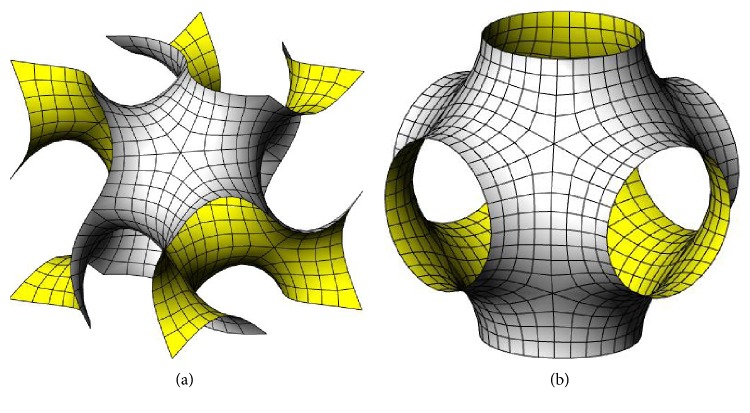
Two types of triply periodic minimal surfaces: (a) Schoen's gyroid and (b) Schwarz's primitive.

Moreover, cellular materials can differ in the geometry of the unit cell. Simple cubic (SC) [22] (Figure 4(a)), body-centered cubic (BCC) [22] (Figure 4(b)), and reinforced body-centered cubic (RBCC) [22] (Figure 4(c)) all come from the same *cubic* cell, with an increasing number of beams. The *octet-truss* (OT) cell [23] (Figure 4(d)) comes from the face-centered cell, and its properties are deeply analyzed in [24]; in particular, it is highlighted how the stiffness and strength of an octet-truss lattice material exceed the corresponding values for metallic foams by a factor between 3 and 10. Other frequently used cells are the modified Gibson-Ashby (GA) [25] (Figure 4(e)) and the modified Wallach-Gibson (WG) cells [26] (Figure 4(f)). Another class of shapes is represented by *triply periodic minimal surfaces* (TPMS), which are periodic surfaces with cubic symmetry and with zero mean curvature that minimize the surface area for given boundary conditions [27] and found application in additive manufacturing [28–30]; Schoen's gyroid (Figure 5(a)), Schwarz P surface (Figure 5(b)) and D surface, and Neovius C(P) surface are TPMS examples [31].

**Figure 6:**
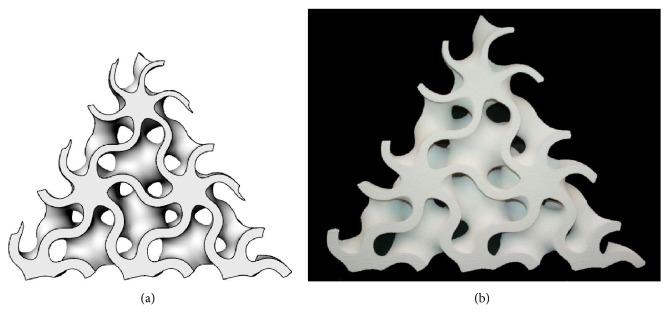
Oblique section of a variable thickness gyroid: (a) virtual and (b) physical model manufactured by additive manufacturing technology (ZPrinter 450 by 3D System).

Finally, focusing on the dimension (i.e., the thickness of a TPMS or beam diameter of a lattice structure) of the cell elements, another sorting is presented. If all the elements of the cell have the same thickness, it can be called *homogeneous cell*; otherwise, if the thickness of struts is different, the cell can be called *heterogeneous*. If the thickness of the cell elements varies gradually according to a pattern, it can be referred as *gradient cell* cellular material.  Figure 6 shows a 3D-printed gradient cell gyroid model with variable thickness.

### 2.2. Cellular Material Geometric Modeling Approaches

**Figure 7:**
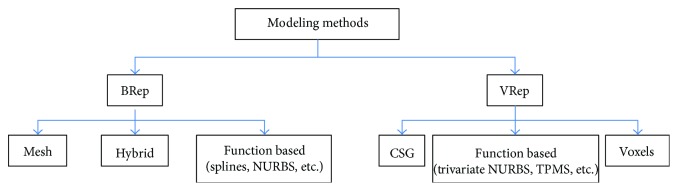
Cellular material modeling methods.

Different methodologies for modeling solid geometries have been proposed in the literature (e.g., [32]) which can be classified into two main groups: boundary representation (BRep) describing the surface between a solid and the surrounding environment and volume representation (VRep) describing the whole solid point by point (Figure 7).

BRep approaches can be divided into two families: discrete, in which the surface is described by polygon mesh, and continuous, in which the surface is described by a mathematical function. Meshes are often used in visualization, rendering, reverse engineering, sculpturing, and additive manufacturing, exchanging data by .stl or .ply file format. On the other hand, the last 60 years saw the evolution of the mathematical definitions of surfaces, which are the foundations of the computer-aided geometric modeling such as Bernstein polynomials [33], Bézier surface [34–36], B-spline, NURBS [37], and subdivision algorithms [38, 39]. These approaches are mainly devoted to freeform surface modeling in a number of applications: automotive, shipbuilding, aerospace, hull, industrial design, and so on.

The constructive solid geometry (CSG) scheme defines complex solids as Boolean operators (union, intersection, and subtraction) of elementary geometry and has found its main application in mechanical geometric modeling [32]. A solid can be described also point by point, adopting function-based approaches such as parametric solid or trivariate NURBS, used in finite element analysis to reduce the preprocessing time [40] or in warping of virtual models. As in surface description, discrete methods can be found in solid representation. As an example, the reconstruction of 3D medical images derived from computed tomography or magnetic resonance imaging is based on voxel (volumetric picture element). The evolution of this approach is the more sophisticated octrees, based on the recursive subdivision of a cubic element [32].

Due to the huge number of elements, the shape complexity, and the multiscale characteristics, these methods need adaptations to be suitable for cellular material geometric modeling. Moreover, although the interest for additive manufacturing has enormously increased in the last years, the capability of commercial CAD software in modeling objects constituted by cellular structures is still limited [8].

Using ACIS [41] as the geometric modeling kernel, Wang et al. [42] developed a hybrid geometric modeling method for conformal cellular structures. Instead of modeling the entire structure using BReps and then converting it to STL, this method creates a STL model of each singular structure and then it joins all the STL models together in order to obtain the whole structure. This allows to reduce computational time. Then, they added a sphere to each node to smooth the geometry and to avoid nonmanifold geometry. Starting from Wang et al.'s results, Chen [43] developed a universal structure generating system. The structure configuration is defined in an XML file, in which the type and all the data of nodes and struts are contained; the XML file is then inputted to a mesh-based structure generating system. A filleting operation is performed too, where the fillet is modeled by a combination of two offsetting operations [44], using the Minkowski sum and difference of two sets [45]. The work is further improved [46], where a space warp is introduced to satisfy design requirements such as having structures with smaller and thicker cells where needed. After the structure is created using the XML file, the space is warped by minimizing an energy function. The possibility of generating internal cellular structures into a generic design space is introduced too.

Savio et al. proposed a modeling and optimization method to design regular cellular structures in [47], where an optimized model is obtained in order to reach the desired utilization for each element. The optimized geometrical model is then modeled using a cylinder with spherical caps, also known as spherinder [48], around each line of the wire model (BReps). Different cell types are analyzed, and for the single cubic one, a new procedure is proposed [49] which avoids NURBS modeling, using a mesh model successively smoothed by the Catmull-Clark subdivision method [38], reducing the stress concentration and increasing the fatigue life. Other subdivision schemes and a generalization of the mesh method to other types of cell are going to be presented, overcoming critical issues on complex models highlighted in literature, such as scalability, robustness, and automation.

**Figure 8:**
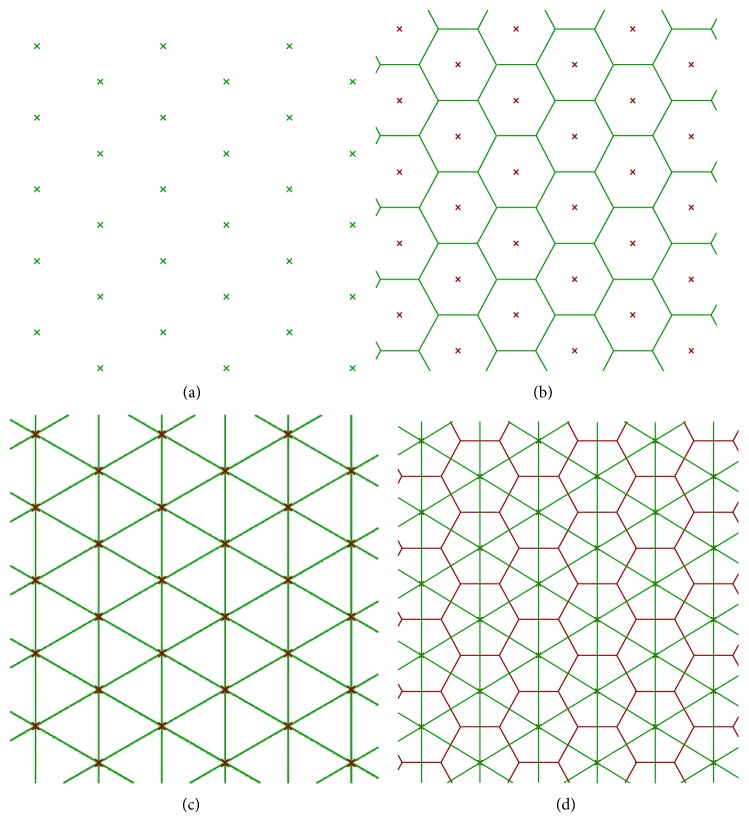
Cell complex dual: (a) regular seed, (b) primal cell complex (Voronoi), (c) dual cell complex (Delaunay), and (d) primal and dual cell complexes together.

Another mesh modeling algorithm is proposed by Medeiros e Sá et al. in [50]. They started from a cell complex inside the volume of the model and then computed the cell complex dual to produce a 3D-printable mesh; the dual of a cell complex is obtained by connecting the central point of two adjacent cells as shown in Figure 8.

**Figure 9:**
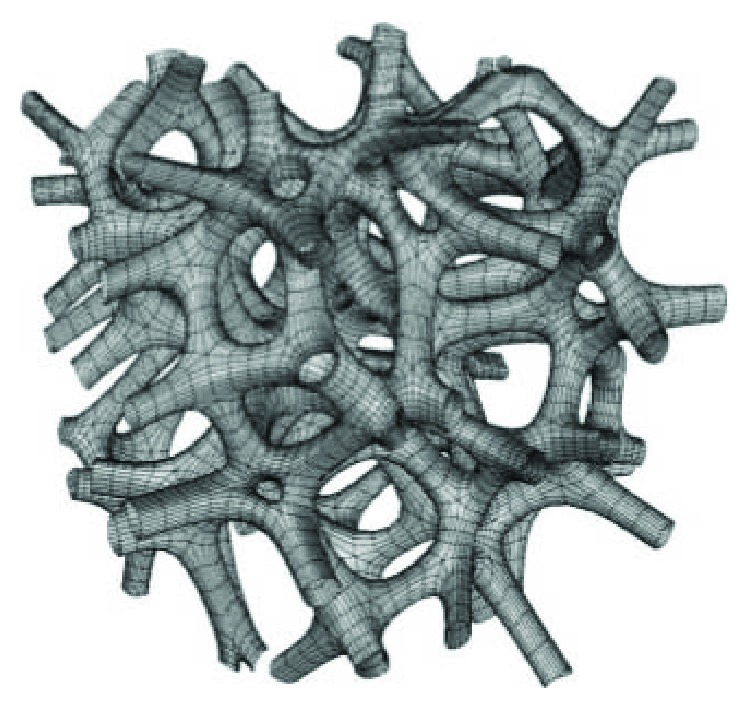
Random cellular structure based on 3D Voronoi tessellation and Catmull-Clark subdivision.

As stated in the previous section, cellular materials can be obtained starting from Voronoi diagrams. Extending the 2D Voronoi diagram concept to 3D, a random lattice can be obtained scaling the Voronoi regions, connecting the adjacent edges and applying Catmull-Clark subdivision [38] as shown in Figure 9 [51–53]. Kou and Tan [54] used the Voronoi diagram and exploited the vertices of the polygons as control points of closed B-spline curves; they also modeled functionally graded structures both scaling B-spline curves gradually as a function of the position and generating Voronoi seeds according to a PDF (probability density function). Chow et al. [55] organized Voronoi seeds in concentric rings, and each seed can be a solid or a void point, generating respectively solid regions or void regions; from the 2D structure, a 3D one is obtained expanding the time dimension of the dynamic pattern in the third dimension of the 2D plane. Fantini et al. [53] used the CAD 3D software Rhinoceros with its plug-in Grasshopper to design scaffolds for bone tissue engineering starting from the patient bone geometry and obtaining a porous structure; their work also aimed to correlate the input parameters, including the number of seeds, with the target ones that are the percentage porosity of the structure and the pore size. This generative design process was further proven by fabricating a sample of Ti6Al4V and morphologically analyzing it by a SEM and a high-resolution micro-CT [56].

Moving on the VRep (volume representation), it is possible to model lattice foam structures taking advantage of CSG (constructive solid geometry [57]). Zeinalabedini et al. [19] obtained a foam by subtracting an ensemble of overlapping elementary spheres from a bulk volume. In a similar way, Gagliardi et al. [58] exploited the Boolean subtraction for the geometric modeling and finite element analyses of a foam structure. Ceruti et al. [59] presented LWSM (lightweight structure modelling), a CAD tool that uses Boolean operations for the modelling of lightweight and lattice structures.

A limit of commercial CAD software is their approach to model microstructures using surface modeling with operations based on BRep. According to Pasko et al. [60], this approach presents both quantitative and qualitative problems. Tasks such as blends, Boolean operations, rendering, and visualization require significant computational resources and large amount of physical memory; Boolean operation failures due to element overlapping issues are likely to happen. Moreover, many techniques for modeling solids are limited because they do not represent the interior of the solid [32]. Assuming the internal homogeneity of the model, they cannot represent internal properties and consequently they are not adequate for modeling functionally graded materials [61]. To overcome these limits, the scientific community has presented different solutions since the early 2000s.

**Figure 10:**
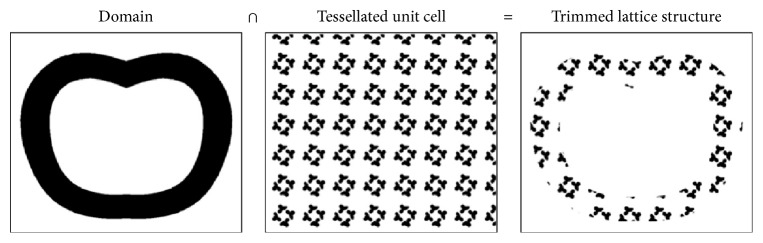
Generation of a trimmed structure by Boolean intersection [63].

A possibility to represent internal properties point by point can be reached defining trivariate functions, and the structure exists only for points that return positive or zero function values, where a zero value indicates the boundary. Pasko et al. [60] used this approach in their work in order to model both regular and irregular microstructures applying particular classes of FRep (function representation) operations defined by R-functions [62] to periodic functions; they stated that FRep parametrization provides more precise and coherent models if compared to BRep representation. Moreover, voxel-based methods have been proposed. A voxel (volumetric element) can be compared to the 2D pixel idea, in 3D space; it is indeed the smallest entity that can be addressed in a discretized space. Similarly, to trivariate functions, voxel-based methods can describe element attributes inside the whole volume. Aremu et al. [63] modeled trimmed and functionally graded lattice structures with and without skin. They first design the model and the cellular structure with voxels, and then they obtain the trimmed lattice structure thanks to a Boolean intersection between the two domains (Figure 10); functional grading is possible by controlling the thickness of the structures utilizing a greyscale image. A method that generates a net skin via orthographic voxel projection is proposed too.

The possibility of using voxels to design graded cellular structures is also presented in [64], where an error diffusion dithering method converts a continuous tone image into a binary representation; in particular, the functional grading image comes from a FEA or a density-based topology optimization. Holdstein et al. [65] adopted a voxel-based approach to design scaffolds for cavities in bone microstructure and hole in-filling.

**Figure 11:**
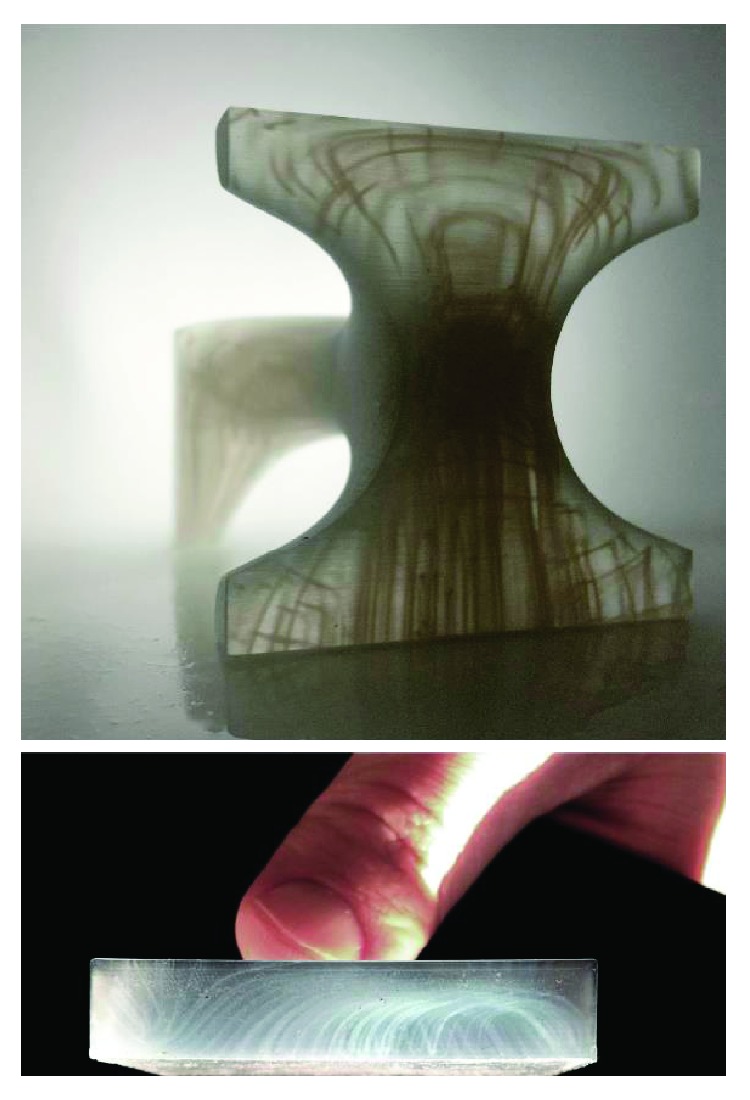
Printed structures modeled with voxel-based method [67].

Due to easy implementation, low requirement of computational resources and physical memory, and robustness and easy way to obtain fillets and available adequate data exchange formats, mesh-based approaches are probably the best solution for describing a cellular material having homogenous characteristics. Volumetric representations, especially voxel methods, seem to be the only approach for modeling functionally graded cellular materials. In this scenario, commercial CAD software is very limited and only few implementation is available. For instance, Figure 11 shows 3D printed-multimaterial models, designed in the voxel modeling software Monolith [66], highlighting the principal stress lines.

## 3. Additive Manufacturing in Scaffold Design for Tissue Engineering

In the last decade, additive manufacturing has gained increasing attention in the biomedical field. According to Lantada and Morgado [68], solutions realized via rapid prototyping can be found in several fields of biomedical engineering. For example, biological and anatomical models and prototypes for diagnosis were one of the first AM exploitations in biomedicine; moreover, the possibility of directly manufacturing implantable devices for soft tissue replacement such as ear and nose is presented. Also, implantable devices for hard tissue replacement and biodevices for tissue engineering are remarkable. Especially in these last two categories, the structure that is going to be implanted, called matrix or scaffold [69, 70], has a key role; examples of scaffold geometries can be found in [71]. In fact, the scaffold has to guarantee biocompatibility [72] and precise mechanical properties depending on the bone that will be replaced and if the structure will be absorbed or not [73]. In order to achieve these results, scaffolds need to have specific properties. Regarding physical properties, porosity, pore size, interconnectivity, and surface finishing are essentials for cell ingrowth and transportation of nutrients and metabolic waste. Wang et al. [16] show disagreement in literature determining the optimal pore size for bone ingrowth; studies have demonstrated how cell ingrowth and vascularization are possible ranging from 30 *μ*m [74] to 900 *μ*m [75] pore size. Bigger-sized pores allow for greater cell ingrowth, while smaller-sized pores can lead to occlusion that prevent liquid exchange and tissue regeneration [76]. Porosity is another important parameter, strictly related to pore size, and has two main purposes. Firstly, a porous scaffold enables nutrients and waste material to diffuse, and secondly, the percentage of porosity can be used to reach the same mechanical properties as the original bone; furthermore, studies [77, 78] showed that highly porous scaffold is not always the right approach for a well-designed scaffold, because even if there is a higher tissue regeneration, biomechanical properties become weak. Also surface finishing and texturing influence tissue ingrowth and fluid dynamics; rough surfaces increase the reaction of osteogenic cells [79] and offer more surface area for integrating [80], while fine-surface finish has to be preferred in joint application, as it reduces friction during motion [81].

Another key feature in a scaffold design is the material. This choice depends on several reasons, such as the function of the scaffold, the implant position in the body, and the scaffold expected life. First and foremost, biocompatibility is fundamental: the scaffold must not generate toxic effects on biological systems. Moreover, if the scaffolds will remain inside the patient body for a long time period, metal alloys are utilized due to the formation of a thin and protective oxide layer that reduces corrosion in vivo [82]. Titanium and its alloys are the most common, but also, cobalt-chromium alloys and stainless steel 316L are widely used. Metals are also utilized for structural application thanks to their excellent mechanical properties. The main issue related to metallic biomaterials concerns their mechanical properties that do not match bone ones; for instance, while Young's modulus of compact bone can range from 10 to 20 GPa [83], Ti6Al4V has a modulus of 100–110 GPa. As previously mentioned, controlling the porosity percentage of the scaffold is a way to reduce and adjust Young's modulus value. Moreover, ceramic-based scaffolds are used because of the similarity to the biological environment. They are hard, brittle, with poor tensile properties, good compression strength, and low frictional properties in articulation [84]. The most common ceramics used in scaffolds are hydroxyapatite (HA) and tricalcium phosphate (TCP): since hydroxyapatite is the most important inorganic constituent of natural hard tissue [85], no biocompatibility problems will arise. Furthermore, biodegradable polymers can be used and they are divided in regulatory approved polymer, like polylactides (PLLA), and nonapproved polymer, like polyorthoester (POE) [72]. In addition, Vert et al. [86] define and distinguish between “biodegradable,” “bioresorbable,” “bioerodible,” and “bioabsorbable” solid polymeric materials. Combining bioactive ceramics with polymers permits to obtain composite material scaffolds with superior mechanical and osteoconductive properties [87, 88] with respect to the singular materials used alone; for instance, Probst et al. [89] designed a polycaprolactone–calcium phosphate scaffold for calvarial reconstruction.

Two other categories of the material utilized for scaffolds are shape memory alloys and hydrogels. The former are capable of recovering their original shape after deformation when stimulated by external environments, and the major representative alloy is nitinol, NiTi, that presents superelasticity and high damping properties too [90]. The problem of NiTi, if used for biomedical purposes, is the presence of Ni that produces allergy; to avoid this drawback, studies have been made to develop surface modification techniques or to use substitution elements, such as Nb instead of Ni [91]. Hydrogels are polymeric networks that absorb water while remaining insoluble and maintaining their structure; these characteristics permit to obtain an environment similar to natural tissues [92].

Concerning the fabrication techniques, two main categories can be identified: conventional techniques and additive manufacturing. Until two decades ago, scaffolds were produced solely through conventional fabrication techniques; fiber bonding, gas foaming, solvent casting, and particulate leaching are some of them, and all have limitations [76, 93, 94]. Extensive use of toxic organic solvents is involved to convert the raw stock into the final scaffold, and this can be both harmful for the patient and time consuming due to the time required for solvent evaporation (days to weeks) [95]. Then, it is difficult (almost impossible) to control the geometry and position of the pores, so the scaffold can be inadequate for the assigned task; modifying process parameters permits to partially control the result, but limits still remain. As already said at the beginning of this section, additive manufacturing is gaining consideration thanks to the possibility of fabricating freeform products, characterized by shape complexity. This perfectly fits the needs in biomedical fields and tissue engineering, where designing scaffolds with controlled size, shape, and porosity distribution becomes possible. AM allows a full customization of the part too, according to the needs of the patient. Different AM technologies have been adopted in order to fabricate biomedical products, and Singh and Ramakrishna [96] wrote a thorough review that analyzes the influence of the type of the AM method and processes parameters on the surface topography, geometrical features, mechanical properties, and biocompatibility in orthopedic applications. Selective laser sintering (SLS) uses a laser beam to selectively sinter the thin layer of powdered materials following the cross-sectional profiles obtained from slicing a 3D-modeled object [97]; both ceramic [98] and polymers [99–101] can be sintered. Similar to SLS, selective laser melting (SLM) uses a laser beam that completely melts powder particles, thus obtaining high-density materials [102]; SLM technique is usually employed with metals, especially titanium alloys [103–105] and cobalt-chromium alloys [106, 107]. Moreover, electron beam melting (EBM) uses an electron beam to melt metal powder in a layer-wise fashion and the process takes place in a vacuum [108–110]. 3D printing (3DP) is based on inkjet printing technology for binding powder materials [111, 112] and is widely used for its simplicity. Lam et al. [113] used 3DP with water as the binder, eliminating the problem of organic solvents; Shanjani et al. [114] fabricated ceramic scaffolds obtaining higher compressive strength and larger pores compared to a sintering technique. A comparison between ceramic scaffold 3D printing and sintering can be also found [115]. Another AM technology is fused deposition modeling [116], where a thermoplastic filament is melted by heating and the semimolten material comes out through a nozzle while it is guided by a 3-axis CNC machine; the apparatus has been modified through the years to minimize costs and permit the use of a wider range of materials, and a compressed air extrusion system [117] and a screw extrusion system [118] were developed.

### 3.1. Geometric Modeling and Design Methods Specific for Scaffolds

Since scaffolds have peculiar requirements regarding cellular materials, specific geometric modeling and design approaches are proposed in the literature.

In order to fabricate scaffolds using AM techniques, a CAD 3D model of the product is needed to obtain the layer-by-layer information necessary to generate tool paths. In particular, a starting point of the model, such as shape and boundaries, is given by the patient's computed tomography (CT) or magnetic resonance imaging (MRI) [71]. These are then used as inputs, and the scaffold is modeled according to the selected parameter values for porosity, pore size, and so on. In the literature, several works for fabricating customized scaffolds have been presented; some concentrate on modeling methods, and others focus on optimizing geometries.

Naing et al. [94] presented a prototype system called computer-aided system for tissue scaffolds (CASTS) that generates .iges and .stl files of the scaffold, ready to be realized through AM, starting from the patient's data collected by imaging technique; the dimension of each unit cell and overall dimensions of the scaffold are considered to obtain the desired porosity and pore size. Singh and Pandey [119] modeled and fabricated a scaffold for a human skull in polyamide PA-2200 starting from MRI/CT scan, and then they did a fitment study obtaining measures through a reverse engineering method. Ambu and Morabito [120] designed scaffolds by repeating both a simple cubic cell and Schwartz's primitive (P) minimal surface; for the P-surface, different porosities were obtained varying the offset value of the surface and the models were numerically evaluated by means of FEA. The P-surface is analyzed in [18] too. Fantini et al. [28] modeled TPMS (diamond and gyroid surfaces) inside a generative design process in order to create a scaffold that meets the input data that are the target pore size, the TPMS unit mesh, and the mesh representing the patient bone geometry. The same authors also implemented a generative design procedure for scaffold modeling based on Voronoi lattice [53].

Schroeder et al. [121] modeled porous objects using stochastic functions in combination with density and constructive solid geometry (CSG) method. Challis et al. [122] maximized the modulus and diffusive flux, which is suitable if the biotransport is driven by concentration gradients rather than pressure gradients, at various porosities; then the porosity is chosen to match the modulus of the bone. Kantaros et al. [95] modeled fixed porosity scaffolds using three different designs (different unit cells) and then compared FE analysis results with experimental ones for compressive strength testing, finding good agreement. Boccaccio et al. [123] developed a mechanobiology-based method in order to find the scaffold structure that maximizes the amount of bone generated within the scaffold; the stimulus parameter is utilized [124], and the investigated parameters are the shape of the pores, their spatial distribution, and the number of pore per unit area. This mechanobiological approach is further adopted to optimize porosity distribution in functionally graded scaffolds [125], where four different distribution laws, three loading conditions, and three scaffold Young's moduli are compared. Naddeo et al. [126] presented an algorithm that starting from a 3D CAD geometry it generates a model, ready to be printed, characterized by a space frame with cylindrical beams organized in order to have the fiber oriented according to the boundary conditions; moreover, the beams are resized according to the target input porosity or the input mechanical stiffness. The models have also been printed and tested, and the results have been compared with FEA output. An improvement of this algorithm can be found in [127], where curved beams have been introduced.

## 4. Conclusions

Cellular materials for scaffolds in tissue engineering, fabricated by additive manufacturing technologies, have recently become a very debated issue in the scientific community. In this work, an original classification scheme based on the geometry of cellular materials has been proposed, regarding the distribution of the cells in the whole structure, the cell topology and geometry, and the cell element dimensions. Literature highlights limits in the commercial CAD software for designing cellular materials. Depending on the presence of functionally graded materials, mesh- or voxel-based geometric modeling approach is preferable due to their robustness. Discussing the design of scaffold for tissue engineering, the analyzed literature shows a clear trend in leaving conventional fabrication techniques, such as gas foaming, and moving toward AM techniques to manufacture tailored scaffolds having accurate and controlled biomechanical properties. Several studies that aim at modeling and optimizing scaffolds have been presented, and the results are promising. As regard biomaterials, different choices are possible, depending on the scaffold purpose: biocompatible metal alloys last longer and are suited for bearing high mechanical stress, while ceramics are more fragile and more similar to the biological environment, ensuring biocompatibility; polymeric scaffolds are fabricated easily and are cheaper. Future directions will have to focus on improving scaffold optimization methods according to patient needs and desired properties. The characterization of scaffold biomechanical properties according to AM fabrication technique parameters has to be examined in depth. Moreover, novel alloying systems or composite matching capable of enhancing the mechanical and biological performance of porous scaffolds is demanded as well.

## References

[1] Hopkinson N., Dickens P., Hague R. (2006). *Rapid Manufacturing*.

[2] International Organization for Standardization - American Society for Testing and Materials International (2015). *ISO/ASTM 52900:2015: Additive Manufacturing. General principles. Terminology*.

[3] Lee J., An J., Chua C. K. (2017). Fundamentals and applications of 3D printing for novel materials. *Applied Materials Today*.

[4] Holmström J., Partanen J., Tuomi J. (2010). Rapid manufacturing in the spare parts supply chain: alternative approaches to capacity deployment. *Journal of Manufacturing Technology Management*.

[5] Rosen D. W. (2007). Computer-aided design for additive manufacturing of cellular structures. *Computer-Aided Design and Applications*.

[6] Ponche R., Kerbrat O., Mognol P. (2014). A novel methodology of design for additive manufacturing applied to additive laser manufacturing process. *Robotics and Computer-Integrated Manufacturing*.

[7] Boyard N., Rivette M., Christmann O. A design methodology for parts using additive manufacturing.

[8] Thompson M. K., Moroni G., Vaneker T. (2016). Design for additive manufacturing: trends, opportunities, considerations, and constraints. *CIRP Annals*.

[9] Gibson L. J., Ashby M. F. (2001). Cellular solids.

[10] Tang Y., Zhao Y. F. (2016). A survey of the design methods for additive manufacturing to improve functional performance. *Rapid Prototyping Journal*.

[11] Amin Yavari S., Ahmadi S. M., Wauthle R. (2015). Relationship between unit cell type and porosity and the fatigue behavior of selective laser melted meta-biomaterials. *Journal of the Mechanical Behavior of Biomedical Materials*.

[12] Kshetrimayum R. S. (2005). A brief intro to metamaterials. *IEEE Potentials*.

[13] Tang Y., Kurtz A., Zhao Y. F. (2015). Bidirectional evolutionary structural optimization (BESO) based design method for lattice structure to be fabricated by additive manufacturing. *Computer-Aided Design*.

[14] Deshpande V. S., Ashby M. F., Fleck N. A. (2001). Foam topology: bending versus stretching dominated architectures. *Acta Materialia*.

[15] Wang H. V. (2005). *A Unit Cell Approach for Lightweight Structure and Compliant Mechanism*.

[16] Wang X., Xu S., Zhou S. (2016). Topological design and additive manufacturing of porous metals for bone scaffolds and orthopaedic implants: a review. *Biomaterials*.

[17] Ryan G., Pandit A., Apatsidis D. (2006). Fabrication methods of porous metals for use in orthopaedic applications. *Biomaterials*.

[18] Shin J., Kim S., Jeong D. (2012). Finite element analysis of Schwarz P surface pore geometries for tissue-engineered scaffolds. *Mathematical Problems in Engineering*.

[19] Zeinalabedini H., Yildiz Y. O., Zhang P. (2016). Homogenization of additive manufactured polymeric foams with spherical cells. *Additive Manufacturing*.

[20] Roberts A. P., Garboczi E. J. (2001). Elastic moduli of model random three-dimensional closed-cell cellular solids. *Acta Materialia*.

[21] Okabe A., Boots B., Chiu S. N. (2009). *Spatial Tessellations*.

[22] Luxner M. H., Stampfl J., Pettermann H. E. (2005). Finite element modeling concepts and linear analyses of 3D regular open cell structures. *Journal of Materials Science*.

[23] Fuller R. B. (1961). *Octet truss*.

[24] Deshpande V. S., Fleck N. A., Ashby M. F. (2001). Effective properties of the octet-truss lattice material. *Journal of the Mechanics and Physics of Solids*.

[25] Roberts A. P., Garboczi E. J. (2002). Elastic properties of model random three-dimensional open-cell solids. *Journal of the Mechanics and Physics of Solids*.

[26] Wallach J. C., Gibson L. J. (2001). Mechanical behavior of a three-dimensional truss material. *International Journal of Solids and Structures*.

[27] Lord E. A., Mackay A. L. (2003). Periodic minimal surfaces of cubic symmetry. *Current Science*.

[28] Fantini M., Curto M., De Crescenzio F., Eynard B., Nigrelli V., Oliveri S. M. TPMS for interactive modelling of trabecular scaffolds for bone tissue engineering.

[29] Speirs M., Van Hooreweder B., Van Humbeeck J. (2017). Fatigue behaviour of NiTi shape memory alloy scaffolds produced by SLM, a unit cell design comparison. *Journal of the Mechanical Behavior of Biomedical Materials*.

[30] Al-Ketan O., Al-Rub R. K. A., Rowshan R. (2017). Mechanical properties of a new type of architected interpenetrating phase composite materials. *Advanced Materials Technologies*.

[31] Schoen A. H. (1970). *Infinite Periodic Minimal Surfaces without Self-Intersections*.

[32] Mortenson M. E. (1997). *Geometric Modeling*.

[33] Bernšteın S. (1912). Démonstration du théoreme de Weierstrass fondée sur le calcul des probabilities. *Communications de la Société Mathématique de Kharkow*.

[34] Bezier P. (1966). Definition numrique des courbes et surfaces I. *Automatisme*.

[35] Bezier P. (1967). Definition numérique des courbes et surfâces II. *Automatisme*.

[36] Bezier P. (1970). *Numerical Control: Mathematics and Applications*.

[37] Piegl L., Tiller W. (1997). *The NURBS Book*.

[38] Catmull E., Clark J. (1978). Recursively generated B-spline surfaces on arbitrary topological meshes. *Computer-Aided Design*.

[39] Doo D., Sabin M. (1978). Behaviour of recursive division surfaces near extraordinary points. *Computer-Aided Design*.

[40] Cottrell J. A., Hughes T. J., Bazilevs Y. (2009). *Isogeometric Analysis: Toward Integration of CAD and FEA*.

[41] Corney J. (1997). *3D Modeling Using the ACIS Kernel and Toolkit*.

[42] Wang H., Chen Y., Rosen D. W. A hybrid geometric modeling method for large scale conformal cellular structures.

[43] Chen Y. A mesh-based geometric modeling method for general structures.

[44] Rossignac J. R., Requicha A. A. G. (1986). Offsetting operations in solid modelling. *Computer Aided Geometric Design*.

[45] Hadwiger H. (1950). Minkowskische addition und subtraktion beliebiger punktmengen und die theoreme von Erhard Schmidt. *Mathematische Zeitschrift*.

[46] Chen Y. (2007). 3D texture mapping for rapid manufacturing. *Computer-Aided Design and Applications*.

[47] Savio G., Gaggi F., Meneghello R. Design method and taxonomy of optimized regular cellular structures for additive manufacturing technologies.

[48] Eusebeia *The Sperical Cylinder*.

[49] Savio G., Meneghello R., Concheri G., Eynard B., Nigrelli V., Oliveri S. M. Optimization of lattice structures for additive manufacturing technologies.

[50] Medeiros e Sá A., Rodriguez Echavarria K., Arnold D. (2014). Dual joints for 3D-structures. *The Visual Computer*.

[51] Piker D. Skeletal mesh. http://www.grasshopper3d.com/forum/topics/skeletal-mesh?id=2985220%3ATopic%3A557380andpage=1#comments.

[52] Co-de-It Vorospace. http://www.co-de-it.com/wordpress/code/grasshopper-code.

[53] Fantini M., Curto M., De Crescenzio F. (2016). A method to design biomimetic scaffolds for bone tissue engineering based on Voronoi lattices. *Virtual and Physical Prototyping*.

[54] Kou X. Y., Tan S. T. (2012). Microstructural modelling of functionally graded materials using stochastic Voronoi diagram and B-spline representations. *International Journal of Computer Integrated Manufacturing*.

[55] Chow H. N., Tan S. T., Sze W. S. (2007). Layered modeling of porous structures with Voronoi diagrams. *Computer-Aided Design and Applications*.

[56] Fantini M., Curto M. (2017). Interactive design and manufacturing of a Voronoi-based biomimetic bone scaffold for morphological characterization. *International Journal on Interactive Design and Manufacturing (IJIDeM)*.

[57] Requicha A. A., Voelcker H. B. (1977). *Constructive Solid Geometry*.

[58] Gagliardi F., De Napoli L., Filice L. (2009). A comparison among FE models to simulate metallic foams forming – an experimental validation. *Materials & Design*.

[59] Ceruti A., Ferrari R., Liverani A., Campana G., Howlett R. J., Setchi R. Design for additive manufacturing using LSWM: a CAD tool for the modelling of lightweight and lattice structures.

[60] Pasko A., Fryazinov O., Vilbrandt T. (2011). Procedural function-based modelling of volumetric microstructures. *Graphical Models*.

[61] Grujicic M., Cao G., Fadel G. M. (2001). Effective materials properties: determination and application in mechanical design and optimization. *Proceedings of the IMechE*.

[62] Shapiro V. (1991). *Theory of R-Functions and Applications: A Primer*.

[63] Aremu A. O., Brennan-Craddock J. P. J., Panesar A. (2017). A voxel-based method of constructing and skinning conformal and functionally graded lattice structures suitable for additive manufacturing. *Additive Manufacturing*.

[64] Brackett D. J., Ashcroft I. A., Wildman R. D. (2014). An error diffusion based method to generate functionally graded cellular structures. *Computers & Structures*.

[65] Holdstein Y., Fischer A., Podshivalov L. Volumetric texture synthesis of bone micro-structure as a base for scaffold design.

[66] Payne A. O., Michalatos P. Monolith. http://www.monolith.zone/.

[67] Payne A. O., Michalatos Autodesk: Project Monolith. https://static1.squarespace.com/static/54450658e4b015161cd030cd/t/56ae214afd5d08a9013c99c0/1454252370968/Monolith_UserGuide.pdf.

[68] Lantada A. D., Morgado P. L. (2012). Rapid prototyping for biomedical engineering: current capabilities and challenges. *Annual Review of Biomedical Engineering*.

[69] Vacanti J. P., Morse M. A., Saltzman W. M. (1988). Selective cell transplantation using bioabsorbable artificial polymers as matrices. *Journal of Pediatric Surgery*.

[70] Langer R., Vacanti J. (1993). Tissue engineering. *Science*.

[71] Sun W., Starly B., Nam J. (2005). Bio-CAD modeling and its applications in computer-aided tissue engineering. *Computer-Aided Design*.

[72] Hutmacher D. W. (2000). Scaffolds in tissue engineering bone and cartilage. *Biomaterials*.

[73] Lin C. Y., Kikuchi N., Hollister S. J. (2004). A novel method for biomaterial scaffold internal architecture design to match bone elastic properties with desired porosity. *Journal of Biomechanics*.

[74] van Tienen T. G., Heijkants R. G. J. C., Buma P. (2002). Tissue ingrowth and degradation of two biodegradable porous polymers with different porosities and pore sizes. *Biomaterials*.

[75] Taniguchi N., Fujibayashi S., Takemoto M. (2016). Effect of pore size on bone ingrowth into porous titanium implants fabricated by additive manufacturing: an *in vivo* experiment. *Materials Science and Engineering: C*.

[76] Leong K. F., Cheah C. M., Chua C. K. (2003). Solid freeform fabrication of three-dimensional scaffolds for engineering replacement tissues and organs. *Biomaterials*.

[77] Bhatia S. N., Chen C. S. (1999). Tissue engineering at the micro-scale. *Biomedical Microdevices*.

[78] Bruder S. P., Kraus K. H., Goldberg V. M. (1998). Critical-sized canine segmental femoral defects are healed by autologous mesenchymal stem cell therapy. *Transactions of the Annual Meeting-Orthopaedic Research Society*.

[79] Albrektsson T., Wennerberg A. (2004). Oral implant surfaces: part 1--review focusing on topographic and chemical properties of different surfaces and in vivo responses to them. *International Journal of Prosthodontics*.

[80] Albrektsson T., Berglundh T., Lindhe J. (2003). Osseointegration: historic background and current concepts. *Clinical Periodontology and Implant Dentistry*.

[81] Singh R., Singh S., Kapoor P. (2016). Investigating the surface roughness of implant prepared by combining fused deposition modeling and investment casting. *Proceedings of the Institution of Mechanical Engineers, Part E: Journal of Process Mechanical Engineering*.

[82] Breme H., Biehl V., Reger N., Murphy W., Black J., Hastings G. (2016). Chapter 1a metallic biomaterials: introduction. *Handbook of Biomaterial Properties*.

[83] Currey J. D. (2002). *Bones: Structure and Mechanics*.

[84] Hutmacher D. W., Schantz J. T., Lam C. X. F. (2007). State of the art and future directions of scaffold-based bone engineering from a biomaterials perspective. *Journal of Tissue Engineering and Regenerative Medicine*.

[85] Tarik Arafat M., Gibson I., Li X. (2014). State of the art and future direction of additive manufactured scaffolds-based bone tissue engineering. *Rapid Prototyping Journal*.

[86] Vert M., Li S. M., Spenlehauer G. (1992). Bioresorbability and biocompatibility of aliphatic polyesters. *Journal of Materials Science: Materials in Medicine*.

[87] Marra K. G., Szem J. W., Kumta P. N. (1999). *In vitro* analysis of biodegradable polymer blend/hydroxyapatite composites for bone tissue engineering. *Journal of Biomedical Materials Research*.

[88] Ignjatović N., Tomić S., Dakić M. (1999). Synthesis and properties of hydroxyapatite/poly-L-lactide composite biomaterials. *Biomaterials*.

[89] Probst F. A., Hutmacher D. W., Müller D. F. (2010). Calvarial reconstruction by customized bioactive implant. *Handchir Mikrochir Plast Chir*.

[90] Castleman L. S., Motzkin S. M., Alicandri F. P. (1976). Biocompatibility of nitinol alloy as an implant material. *Journal of Biomedical Materials Research Part A*.

[91] Miyazaki S., Kim H. Y., Hosoda H. (2006). Development and characterization of Ni-free Ti-base shape memory and superelastic alloys. *Materials Science and Engineering: A*.

[92] Melchels F. P. W., Domingos M. A. N., Klein T. J. (2012). Additive manufacturing of tissues and organs. *Progress in Polymer Science*.

[93] Cheah C. M., Chua C. K., Leong K. F. (2003). Development of a tissue engineering scaffold structure library for rapid prototyping. Part 1: investigation and classification. *The International Journal of Advanced Manufacturing Technology*.

[94] Naing M. W., Chua C. K., Leong K. F. (2005). Fabrication of customised scaffolds using computer-aided design and rapid prototyping techniques. *Rapid Prototyping Journal*.

[95] Kantaros A., Chatzidai N., Karalekas D. (2016). 3D printing-assisted design of scaffold structures. *The International Journal of Advanced Manufacturing Technology*.

[96] Singh S., Ramakrishna S. (2017). Biomedical applications of additive manufacturing: present and future. *Current Opinion in Biomedical Engineering*.

[97] Paul B. K., Baskaran S. (1996). Issues in fabricating manufacturing tooling using powder-based additive freeform fabrication. *Journal of Materials Processing Technology*.

[98] Vail N. K., Swain L. D., Fox W. C. (1999). Materials for biomedical applications. *Materials & Design*.

[99] Tan K. H., Chua C. K., Leong K. F. (2003). Scaffold development using selective laser sintering of polyetheretherketone–hydroxyapatite biocomposite blends. *Biomaterials*.

[100] Simpson R. L., Wiria F. E., Amis A. A. (2008). Development of a 95/5 poly (L-lactide-*co*-glycolide)/hydroxylapatite and *β*-tricalcium phosphate scaffold as bone replacement material via selective laser sintering. *Journal of Biomedical Materials Research Part B: Applied Biomaterials*.

[101] Zhou W. Y., Lee S. H., Wang M. (2008). Selective laser sintering of porous tissue engineering scaffolds from poly (L-lactide)/carbonated hydroxyapatite nanocomposite microspheres. *Journal of Materials Science: Materials in Medicine*.

[102] Kruth J. P., Froyen L., Van Vaerenbergh J. (2004). Selective laser melting of iron-based powder. *Journal of Materials Processing Technology*.

[103] Van Bael S., Chai Y. C., Truscello S. (2012). The effect of pore geometry on the in vitro biological behavior of human periosteum-derived cells seeded on selective laser-melted Ti6Al4V bone scaffolds. *Acta Biomaterialia*.

[104] Van der Stok J., van der Jagt O. P., Amin Yavari S. (2013). Selective laser melting-produced porous titanium scaffolds regenerate bone in critical size cortical bone defects. *Journal of Orthopaedic Research*.

[105] Yan C., Hao L., Hussein A. (2015). Ti–6Al–4V triply periodic minimal surface structures for bone implants fabricated via selective laser melting. *Journal of the Mechanical Behavior of Biomedical Materials*.

[106] Vandenbroucke B., Kruth J.-P. (2007). Selective laser melting of biocompatible metals for rapid manufacturing of medical parts. *Rapid Prototyping Journal*.

[107] Wu L., Zhu H., Gai X. (2014). Evaluation of the mechanical properties and porcelain bond strength of cobalt-chromium dental alloy fabricated by selective laser melting. *The Journal of Prosthetic Dentistry*.

[108] Heinl P., Müller L., Körner C. (2008). Cellular Ti–6Al–4V structures with interconnected macro porosity for bone implants fabricated by selective electron beam melting. *Acta Biomaterialia*.

[109] Ponader S., von Wilmowsky C., Widenmayer M. (2010). *In vivo* performance of selective electron beam-melted Ti-6Al-4V structures. *Journal of Biomedical Materials Research Part A*.

[110] Parthasarathy J., Starly B., Raman S. (2010). Mechanical evaluation of porous titanium (Ti6Al4V) structures with electron beam melting (EBM). *Journal of the Mechanical Behavior of Biomedical Materials*.

[111] Sachs E. M., Haggerty J. S., Cima M. J. (1993). *Three-Dimensional Printing Techniques*.

[112] Brunello G., Sivolella S., Meneghello R. (2016). Powder-based 3D printing for bone tissue engineering. *Biotechnology Advances*.

[113] Lam C. X. F., Mo X. M., Teoh S. H., Hutmacher D. W. (2002). Scaffold development using 3D printing with a starch-based polymer. *Materials Science and Engineering: C*.

[114] Shanjani Y., Croos D., Amritha J. N. (2010). Solid freeform fabrication and characterization of porous calcium polyphosphate structures for tissue engineering purposes. *Journal of Biomedical Materials Research Part B: Applied Biomaterials*.

[115] Warnke P. H., Seitz H., Warnke F. (2010). Ceramic scaffolds produced by computer-assisted 3D printing and sintering: characterization and biocompatibility investigations. *Journal of Biomedical Materials Research Part B: Applied Biomaterials*.

[116] Crump S. S. (1992). *Apparatus and Method for Creating Three-Dimensional Objects*.

[117] Hoque M. E., San W. Y., Wei F. (2009). Processing of polycaprolactone and polycaprolactone-based copolymers into 3D scaffolds, and their cellular responses. *Tissue Engineering Part A*.

[118] Shor L., Güçeri S., Wen X. (2007). Fabrication of three-dimensional polycaprolactone/hydroxyapatite tissue scaffolds and osteoblast-scaffold interactions *in vitro*. *Biomaterials*.

[119] Singh J. P., Pandey P. M. (2013). Fitment study of porous polyamide scaffolds fabricated from selective laser sintering. *Procedia Engineering*.

[120] Ambu R., Morabito A. E., Eynard B., Nigrelli V., Oliveri S. M. Design and analysis of tissue engineering scaffolds based on open porous non-stochastic cells.

[121] Schroeder C., Regli W. C., Shokoufandeh A. (2005). Computer-aided design of porous artifacts. *Computer-Aided Design*.

[122] Challis V. J., Roberts A. P., Grotowski J. F. (2010). Prototypes for bone implant scaffolds designed via topology optimization and manufactured by solid freeform fabrication. *Advanced Engineering Materials*.

[123] Boccaccio A., Uva A. E., Fiorentino M. (2016). A mechanobiology-based algorithm to optimize the microstructure geometry of bone tissue scaffolds. *International Journal of Biological Sciences*.

[124] Prendergast P. J., Huiskes R., Søballe K. (1997). Biophysical stimuli on cells during tissue differentiation at implant interfaces. *Journal of Biomechanics*.

[125] Boccaccio A., Uva A. E., Fiorentino M. (2016). Geometry design optimization of functionally graded scaffolds for bone tissue engineering: a mechanobiological approach. *PloS One*.

[126] Naddeo F., Cappetti N., Naddeo A. (2017). Novel “load adaptive algorithm based” procedure for 3D printing of cancellous bone-inspired structures. *Composites Part B: Engineering*.

[127] Naddeo F., Naddeo A., Cappetti N. (2017). Novel “load adaptive algorithm based” procedure for 3D printing of lattice-based components showing parametric curved micro-beams. *Composites Part B: Engineering*.

